# Ethiopathogenic mechanisms of endometriosis-related infertility

**DOI:** 10.5935/1518-0557.20190029

**Published:** 2019

**Authors:** Michele Gomes Da Broi, Rui Alberto Ferriani, Paula Andrea Navarro

**Affiliations:** 1 Human Reproduction Division, Department of Obstetrics and Gynecology, Ribeirão Preto School of Medicine, University of São Paulo, Ribeirão Preto, SP, Brazil; 2 National Institute of Hormones and Woman’s Health, CNPq, Brazil

**Keywords:** endometriosis, infertility, etiopathogenesis

## Abstract

Endometriosis is a highly prevalent disease among women of reproductive age and is frequently associated to infertility. However, the mechanisms underlying endometriosis-related infertility are still not completely known. Several studies have been conducted in order to elucidate this question. Besides anatomical changes that may impair gametes and embryo transport along the tubes; a smaller ovarian reserve due to advanced endometriosis and endometriomas; and a dysregulated hypothalamic-pituitary-ovarian axis, there are pieces of evidence suggesting that the peritoneal ectopic endometrial foci may induce a local inflammatory response, with the recruitment of macrophages, cytokine release, and reactive oxygen species generation, leading to a pro-oxidant peritoneal microenvironment. These alterations may be systemically reflected and also affect the follicular microenvironment. A harmful follicular fluid may disrupt cumulus cells functions and, consequently, compromise oocyte competence. There is also evidence suggesting that the peritoneal fluid of women with endometriosis may alter sperm function. Reduced endometrial receptivity is also pointed as a possible mechanism involved in endometriosis-related infertility, which needs further investigation.

## INTRODUCTION

Endometriosis is a disease defined as the presence of endometrial tissue outside the uterine cavity (Burney & Giudice, 2012; Gupta *et al*., 2006). It is highly prevalent among women of reproductive age (Burney & Giudice, 2012), which is very alarming, since endometriosis is also frequently associated to infertility (ASRM, 2012). It affects approximately 25 to 50% of infertile women, and 30 to 50% of endometriosis patients have difficulties to become pregnant (ASRM, 2012). Although the literature widely addresses the association between the disease and infertility (Akande *et al*., 2004; Carvalho *et al*., 2012; Da Broi & Navarro, 2016b; Gupta *et al*., 2008; Marcoux *et al*., 1997; Parazzini, 1999), the etiopathogenic mechanisms involved in this relation have not yet been fully understood.

Here, we review and discuss on the role of some possible mechanisms underlying this condition, including anatomical changes of the reproductive tract and smaller ovarian reserve possibly involved in advanced disease infertility, and also the role of peritoneal and follicular microenvironments, cumulus cells (CC), sperm function, and endometrial receptivity as possible mechanisms involved in the fertility impairment in patients with early endometriosis.

### Endometriosis-related infertility

Although endometriosis is frequently associated to infertility (ASRM, 2012), the mechanisms underlying this condition are still not completely known. Several studies have been conducted in order to elucidate this question, and authors have suggested different mechanisms potentially involved in infertility impairment, including anatomical and microenvironmental conditions that may negatively impact the oocyte competence acquisition, egg fertilization, zygote transport within the tube and embryo implantation.

In cases of advanced disease (rAFS III and IV), anatomical changes of the reproductive tract such as peritubal and periovarian adhesions and pelvic distortions are indicated as limiting factors, which could impair the oocyte capture by the fimbriae, its passage through the tuba, as well as the gametic interaction and the embryonic path to the uterine cavity (ASRM, 2012; Catenacci & Falcone, 2008; Schenken *et al*., 1984). It has also been suggested a smaller ovarian reserve in women with advanced endometriosis (Seyhan *et al*., 2015), especially in cases of endometrioma (Hock *et al*., 2001; Sanchez *et al*., 2014; Uncu *et al*., 2013). In this sense, some authors defend that ovarian endometrioma *per* se may affect ovarian reserve (Goodman *et al*., 2016; Uncu *et al*., 2013). It is believed that ovarian tissue may be target of toxic substances contained in the endometrioma, which could diffuse in the adjacent tissue and culminate with the reduced ovarian reserve (Sanchez *et al*., 2014). On the other hand, some researchers believe that surgical treatment of endometriomas promotes the damage on ovarian tissue, predisposing to low follicle count (Cranney *et al*., 2017; Goodman *et al*., 2016; Mehdizadeh Kashi *et al*., 2017).

However, infertility presented by women with early endometriosis (rAFS I and II), where pelvic anatomical distortions are not present, raises questions about the involvement of other mechanisms in the impairment of fertility in patients with the disease (Da Broi & Navarro, 2016b; Holoch & Lessey, 2010). In this sense, it is believed that the peritoneal, follicular and endometrial microenvironments are altered in these women, with consequent damages to folliculogenesis, ovulation, oocyte quality, endometrial receptivity and, even, sperm function (Agarwal *et al*., 2012; Gupta *et al*., 2008).

### Peritoneal microenvironment and immune function

Evidence from literature suggest that the immune function is possibly dysregulated in endometriosis patients (Gupta *et al*., 2008; Miller *et al*., 2017). It is questioned if women with endometriosis have immunological dysfunction preventing the removal of endometrial implants and leading to tissue adhesion in the peritoneal cavity (Ahn *et al*., 2015a). It is also believed that peritoneal endometrial lesions are responsible for the activation of macrophages, with consequent increase in the generation of inflammatory factors, reactive oxygen and nitrogen species, cytokines, growth factors, and prostaglandins. A marked inflammatory response, with exacerbation of reactive species and cytokines, would make the pelvic environment adverse, which would be reflected in the peritoneal fluid (PF) of these women (Agarwal *et al*., 2003; Gupta *et al*., 2006; Ruder *et al*., 2008; Szczepańska *et al*., 2003). Corroborating this reasoning, studies have shown changes in the PF composition of women with endometriosis, including changes in cellular and humoral mediators (Cheong *et al*., 2002; Eisermann *et al*., 1988; Jørgensen *et al*., 2017; Keenan *et al*., 1995), including pro-inflammatory cytokines such as tumor necrosis factor (TNF)-α, interleukin (IL)-1β, IL-6, IL-8, IL-10, IL-13, IL-17, IL-33, monocyte chemoattractant protein (MCP)-1, macrophage migration inhibitory factor (MIF) and Regulated on Activation, Normal T Cell Expressed and Secreted (RANTES) (Ahn *et al*., 2015b; Bersinger *et al*., 2006; Harada *et al*., 1997; Punnonen *et al*., 1996; Sikora *et al*., 2012; Wang *et al*., 2018; Yoshino *et al*., 2003), chemokines (Margari *et al*., 2013), angiogenic factors (Ahn *et al*., 2015b; Kianpour *et al*., 2013; Yoshino *et al*., 2003), and increased activated macrophages, T-lymphocytes and natural killer cells (Lebovic *et al*., 2001). These alterations may lead to chronic inflammation, proliferation of lesions, local hormonal imbalance, what may lead to poor oocyte quality, poor sperm motility, embryo toxicity and reduced endometrial receptivity (Miller *et al*., 2017). In addition, there is evidence of altered oxidative stress (OS) markers in the PF of these women (Polak *et al*., 2013; Santulli *et al*., 2015; Shanti *et al*., 1999). As a consequence of these alterations, studies have suggested an adverse effect of PF on the reproductive capacity of the patients (Jianini *et al*., 2017; Gupta *et al*., 2008; Mansour *et al*., 2009a; Mansour *et al*., 2010).

Because the PF bathes the ovaries and maintains direct contact with the oocyte during ovulation and in its initial course through the uterine tube, changes in this microenvironment may culminate in oocyte damage and be involved in the impairment of oocyte quality in endometriosis patients. Accordingly, studies with murine model indicate damage to spindle and chromosomes after incubation of oocytes in metaphase II with PF from women with the disease (Mansour *et al*., 2009a; Mansour *et al*., 2010) which were reduced with the addition of an antioxidant, suggesting the role of OS in promoting the oocyte alterations (Mansour *et al*., 2009a). In addition, in a recent study, meiotic damage to bovine oocytes was evidenced after *in vitro* oocyte maturation in the presence of PF from infertile patients with endometriosis, suggesting changes in this fluid could also compromise oocyte development during maturation and possibly affect oocyte quality of these patients (Jianini *et al*., 2017).

### Follicular microenvironment

Evidences have suggested the occurrence of systemic OS in women with the disease (Andrade *et al*., 2010; Da Broi *et al*., 2016; Liu *et al*., 2013; Nasiri *et al*., 2017; Singh *et al*., 2013), which could consequently reach the ovaries and affect intrafollicular oocyte development, since the ovarian cortex is highly vascularized, especially in the final period of folliculogenesis (Tamanini & De Ambrogi, 2004).

Different studies have investigated changes in the follicular fluid (FF) composition of women with endometriosis, such as cytokines (Singh *et al*., 2016; Wu *et al.*, 2017) OS markers (Choi *et al*., 2015; Da Broi *et al*., 2016a; Huang *et al*., 2014; Liu *et al*., 2013; Nasiri *et al*., 2017; Prieto *et al*., 2012; Singh *et al*., 2013), growth factors (Choi *et al*., 2015), metals (Singh *et al*., 2013), prostaglandins (Du *et al*., 2013), macrophages activation pattern (Lamaita *et al*., 2012), lipidic (Cordeiro *et al*., 2015) and proteic profiles (Lo Turco *et al*., 2013). In this sense, the evidences of OS in the follicular microenvironment of these women (Choi *et al*., 2015; Da Broi *et al*., 2016a; Huang *et al*., 2014; Liu *et al*., 2013; Nasiri *et al*., 2017; Prieto *et al*., 2012; Singh *et al*., 2013), suggest that not only their PF, but also their FF may contain substances harmful to the acquisition of oocyte competence. In this regards, studies evaluating the effect of FF of infertile women with endometriosis on *in vitro* maturation of bovine oocytes showed spindle and chromosomal damage (Da Broi *et al*., 2014), which were prevented by the addition of antioxidants to the maturation medium, suggesting a pro-oxidant microenvironment in the ovarian follicles of these women (Giorgi *et al*., 2016). Possibly, these alterations are consequence of OS damage on oocyte cell structures. Recently, it was evidenced the presence of higher levels of eight-hydroxy-2-deoxyguanosine (8OHdG) in the FF of infertile women with endometriosis, suggesting oxidative DNA damage in cumulus-oocyte complexes, being a possible mechanism involved in the impairment of oocyte quality in these patients (Da Broi *et al*., 2016a).

### Cumulus cells

The CC are considered indirect markers of oocyte quality (Assou *et al*., 2006; Hamamah *et al*., 2006; Hamel *et al*., 2008; Haouzi & Hamamah, 2009), since they are responsible for energetic metabolism (Downs & Utecht, 1999; Monniaux, 2016; Paczkowski *et al*., 2013; Saito *et al*., 1994), ions support (FitzHarris *et al*., 2007), transcriptional maintenance (Albertini *et al*., 2001), maturation (Li & Albertini, 2013; Tanghe *et al*., 2002) and defense (Albertini *et al*., 2001; Lolicato *et al*., 2015; Shaeib *et al.*, 2016; Tanghe *et al*., 2002) of the female gamete, so that changes in these cells can harm follicular development and indicate damage to the oocyte.

Studies comparing the expression of genes related to steroidogenesis, acquisition of oocyte competence, and OS in CC of infertile women with and without endometriosis have been performed. Accordingly, the aromatase-encoding gene (*CYP19A1)* (Barcelos *et al*., 2015; Hosseini *et al*., 2016)*,* and the cyclooxygenase 2 (COX-2)-encoding gene (*PTGS2)* (Donabela *et al*., 2011) that may mediate *CYP19A1* induction, seem to be both lower in CC of infertile women with endometriosis compared to infertile controls undergoing controlled ovarian stimulation for intracytoplasmic sperm injection (ICSI). In this regards, it has been suggested an epigenetic alteration may be involved in *CYP19A1* gene deregulation in CC of these patients (Hosseini *et al*., 2016). Altogether, these data suggest reduced aromatase and, consequently, possibly altered follicular steroidogenesis and impaired oocyte quality in infertile women with endometriosis, what requires confirmation by further studies.

The evaluation of enzymatic antioxidants gene expression in CC of infertile women with and without endometriosis evidenced increased superoxide dismutase 1 (*SOD1*) expression in the moderate/severe endometriosis group compared to women with minimal/mild endometriosis and controls. It suggests that advanced disease may induce pronounced OS and stimulate increased expression of this antioxidant as an attempt to prevent oxidative damage to oocytes (Donabela *et al*., 2015).

Moreover, alterations in mitochondrial function of CC from infertile women with endometriosis have also been suggested as a possible mechanism involved in oocyte damage (Hsu *et al*., 2015). Some authors have also evidenced alterations in CC's cell cycle of infertile women with advanced disease (Toya *et al*., 2000), which may justify the increased apoptosis observed by others in their CC (Díaz-Fontdevila *et al*., 2009) and, consequently, lead to abnormal folliculogenesis in these women (Toya *et al*., 2000).

### Hypothalamic-pituitary-ovarian Axis and Ovarian Function

Likewise, endometriosis has been identified as a disease related to changes in the hypothalamic-pituitary-ovarian axis, with abnormal luteinizing hormone (LH) and prolactin secretion (Cahill & Hull, 2000; Cunha-Filho *et al*., 2001), which may result in ovary dysfunction in women with the disease. Moreover, granulosa cells of infertile women with early endometriosis seem to be less sensitive to LH stimulation (Cahill *et al*., 2003). In this sense, studies point to the occurrence of an abnormal luteal phase (Cunha-Filho *et al*., 2001; 2003; Schenken *et al*., 1984) and a longer follicular phase (Cahill *et al*., 1997) in these patients, what may affect the patterns of estrogen and progesterone secretion (Cahill & Hull, 2000; Cunha-Filho *et al*., 2003). Accordingly, reduced estrogen, androgen and progesterone, and increased activin were found in the follicular fluid of patients with endometriosis (Cahill & Hull, 2000). Consequently, these alterations may, directly or indirectly, damage follicular growth, reduce dominant follicle size, affect follicles maturation, and compromise ovulation in women with endometriosis (Doody *et al*., 1988; Schenken *et al*., 1984; Tummon *et al*., 1988).

### Sperm function

In addition, high growth factors, cytokines, activated macrophages, TNF-α concentrations and OS present in the PF from infertile women with endometriosis may be toxic to sperm function (Aeby *et al*., 1996; Liu *et al*., 2000; Mansour *et al.*, 2009b). These altered factors may induce sperm DNA fragmentation (Mansour *et al*., 2009b), disrupt sperm membrane permeability or integrity (Said *et al*., 2005), reduce sperm motility (Liu *et al*., 2000; Oral *et al*., 1996), impair the interaction between the sperm and the epithelium of the uterine tube (Reeve *et al*., 2005), promote abnormal sperm acrosome reaction (Arumugam, 1994) and impair sperm-oocyte fusion (Aeby *et al*., 1996), representing another possible mechanism involved in endometriosis-related infertility.

### Endometrial microenvironment

Some authors have also considered the role of the endometrium in infertility related to endometriosis, so that alterations in endometrial receptivity due to late histological maturation or biochemical disturbances in the eutopic endometrium may compromise embryo implantation in women with the disease (Bulletti *et al*., 2010; Giudice & Kao, 2004).

Studies suggest that the endometrium may be functionally altered during the implantation window in these patients (Wei *et al*., 2009). Among the molecules identified with aberrant expression during the window of implantation in the eutopic endometrium of women with endometriosis there are receptors of progesterone and estrogen (Young, 2013), integrins (Giudice & Kao, 2004), leukemia inhibitory factor (LIF), glicodelin A, (GdA), osteopontin (OPN), lipolysophosphatidic acid receptor 3 (LPA3), HOXA10 (Revel, 2012), which are related to the establishment of endometrial receptivity and/or to the interaction between the endometrium and the embryo (Giudice *et al*., 2002).

On the other hand, recent studies have discussed the relevance of endometrial factor for endometriosis-related infertility (Broi *et al*., 2017; Da Broi *et al*., 2017; Garcia-Velasco *et al*., 2015). Simultaneous expression of crucial genes for endometrial receptivity does not appear to undergo significant changes in infertile women with endometriosis during the implantation window (Broi *et al*., 2017). Likewise, the presence and stage of development of pinopods, which were once considered classic biomarkers of the implantation window in the human endometrial epithelium (Achache & Revel, 2006; Aghajanova *et al*., 2003; Nikas, 1999; Nikas & Makrigiannakis, 2003; Nikas & Psychoyos, 1997; Xu *et al*., 2012), also appear to be similar in women with the disease and controls (Da Broi *et al*., 2017; Ordi *et al*., 2003).

Recently, Garcia-Velasco *et al*. (2015) published a pilot study in which samples of eutopic endometrium from infertile women with endometriosis and infertile controls were evaluated using a molecular diagnostic tool (ERA), and showed no difference in the expression of the genes predicted for receptivity between the groups.

## CONCLUSIONS

Although the mechanisms involved in endometriosis-related infertility are still not completely understood, some evidences suggest multiple factors that may potentially affect patient's fertility. In addition to the pelvic anatomical alterations likely to compromise the gametic interaction and the altered steroidogenesis, ovulation and disrupted ovarian function, peritoneal changes seem to promote a harmful and pro-oxidative microenvironment, which may compromise the CC and the follicular microenvironment, affecting folliculogenesis and, possibly, the oocyte competence in women with endometriosis. Peritoneal alterations may also damage the spermatozoa and difficult gametes interaction. The role of compromised endometrial receptivity is still controversial; however, recent evidence points to a major role of the oocyte factor in impaired fertility of infertile women with endometriosis.
